# Uncommon presentation: isolated small bowel perforation after blunt abdominal trauma in a pediatric patient; a case report

**DOI:** 10.1097/MS9.0000000000001421

**Published:** 2023-11-01

**Authors:** Shailendra Katwal, Aastha Ghimire, Nishant Joshi

**Affiliations:** a Department of Radiology, Dadeldhura Subregional Hospital, Dadeldhura; b Patan Academy of Health Sciences, Lalitpur, Nepal

**Keywords:** atypical presentation, blunt abdominal trauma, case report, small bowel perforation, surgical intervention

## Abstract

**Introduction and importance:**

A small bowel perforation occurring in isolation as a result of blunt abdominal trauma (BAT) is a rare event, especially in pediatric patients. The unusual presentation and lack of distinct clinical indications can pose a challenge in promptly diagnosing this condition. This study seeks to underscore the importance of early detection and immediate surgical intervention when dealing with small bowel perforations following BAT.

**Case presentation:**

A 13-year-old girl arrived at the emergency department after falling from a cliff. Initial evaluations, including a physical examination and imaging studies, did not uncover any notable irregularities. Nevertheless, the persistent abdominal discomfort and pain prompted further concerns. A contrast-enhanced computed tomography scan was performed, confirming ileal perforation. The patient subsequently underwent exploratory laparotomy, which resulted in successful surgical treatment.

**Clinical discussion:**

BAT causes increased intraluminal pressure, leading to blowout perforation of the small bowel. Diagnosis can be challenging, particularly in the absence of immediate symptoms or conclusive radiographic findings. Close observation and repeated examinations are essential to detect delayed perforations. Early surgical intervention within 12 h of injury has been shown to significantly reduce complications and mortality rates.

**Conclusion:**

Isolated small bowel perforation is a rare occurrence in pediatric patients, and timely diagnosis and surgical intervention are crucial for favorable outcomes. Diagnostic imaging, like contrast-enhanced computerized tomography, helps identify the condition when clinical findings are inconclusive. Comprehensive counseling is essential for patients and their families to understand potential risks and intervention needs, ensuring appropriate management, and treatment delays.

## Introduction

Highlights

- This case report highlights the rarity and diagnostic challenges of isolated small bowel perforation following blunt abdominal trauma, particularly in pediatric patients.
- Timely recognition and surgical intervention are crucial in managing this condition, as delayed diagnosis can lead to complications and adverse outcomes.
- Comprehensive counseling for patients and their families regarding potential risks and the need for further intervention is essential to ensure appropriate management and prompt treatment.

Blunt abdominal trauma (BAT) leads to the injury of intra-abdominal organs among which injuries to solid organs are the most reported ones^1^. Isolated perforation of the small bowel after BAT is infrequent and extremely rare with a reported incidence of 0.3%^1^. The incidence of small bowel injury appears to be further lower in children than in adults^2^. The vast majority of intestinal perforations following BAT is caused by motor vehicle accidents, but can also result from physical assault by human beings or animals, fall from height, or injury caused by a bicycle handlebar^2^. Clinical presentation of small bowel perforation is usually vague and physical examination inconclusive so suspicion comes only when a marked deterioration of the clinical status has been established^3^. Significant factors affecting mortality are therapeutic delay of 24 h or more, and multiple injuries^4^. Here, we present a rare case of isolated ileal perforation following BAT in a 13-year-old girl who underwent successful surgical treatment but whose diagnosis was eluded by atypical symptoms.

This case report has been reported in accordance with the CARE criteria^5^.

### Case details

A 13-year-old girl was brought by her parents to our hospital’s emergency department after allegedly falling from a cliff that was ~13 feet in height. The patient resided in the rural hills of Nepal and reportedly fell while playing with her friend, which aligned with the injury mechanism described by the patient. The initial primary survey yielded unremarkable results. Subsequently, the Focused Assessment with Sonography for Trauma (FAST) scan showed normal findings. Her blood pressure measured 90/60 mmHg, her pulse rate was 95 beats per minute, her respiratory rate was 20 breaths per minute, and her oxygen saturation on room air was 93%. During the secondary survey, notable findings included a 2 cm×2 cm laceration located on the left supraorbital area, multiple abrasions over the periumbilical, suprapubic, right and left iliac fossa of the abdomen, and a 3 cm×2 cm laceration on the left lumbar region. The patient reported pain in these injury sites, as well as generalized abdominal discomfort. The patient denied experiencing headaches, nausea, vomiting, chest pain, shortness of breath, or pain in any of her limbs. She had no difficulty passing urine. The systemic examination yielded normal results. Her abdomen was soft and showed no signs of distension or tenderness. There were no injuries inconsistent with the reported mechanism of injury, and the patient exhibited no abnormal behavior.

**Figure 1:**
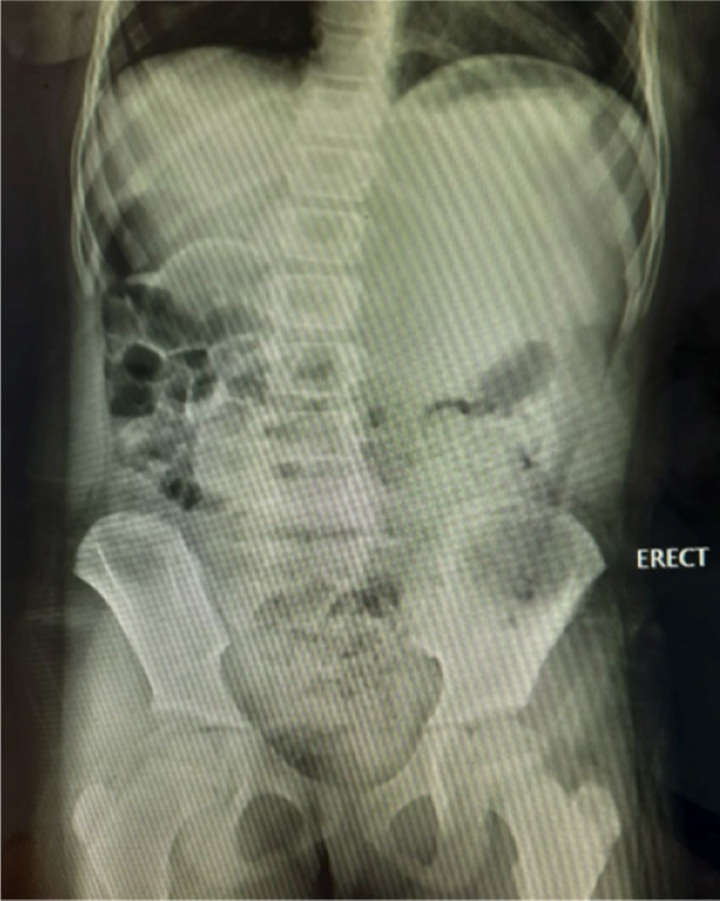
Erect Radiograph of the abdomen showing no intraperitoneal free air.

**Figure 2:**
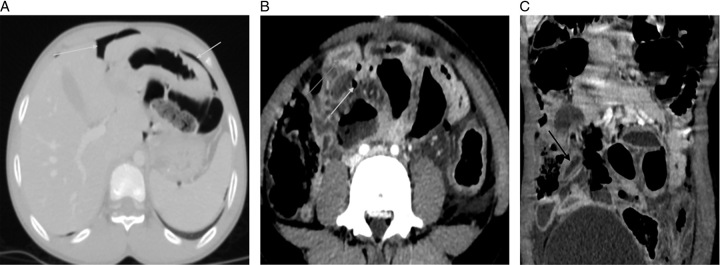
A, Lung window axial CECT image of the abdomen at the hepatic level showing free in the perigastric regions (white arrows). B, Contrast-enhanced axial image of the abdomen showing a thickened wall of the terminal ileum (gray arrow) with suspicious wall defect and air foci in terminal ileum (white arrow). C, Coronal contrast-enhanced CT image showing the thickened bowel loops in the distal ileum (black arrow).

The patient was admitted to the emergency department for further evaluation. She was monitored at regular intervals for any new symptoms and any changes in her vital parameters. A complete blood count, renal function test, serum electrolytes and urine for routine examination, and radiography of the chest and abdomen were ordered (Table 1). The X-rays revealed no abnormalities (Fig. 1). After receiving preliminary care for her external injuries she was then admitted to the inpatient ward of the hospital for observation and was administered appropriate analgesics and intravenous fluids. The patient at admission complained of no other symptoms apart from abdominal discomfort and pain over the right lower abdomen. The patient remained stable throughout the night, with no changes in her symptoms or signs. However, the discomfort and pain she experienced persisted. In addition to repeating the complete blood count blood tests, further investigations were conducted; including serum amylase, serum lipase, and C-reactive protein level (Table 1). Due to the patient’s elevated acute phase reactants and persistent discomfort, a mere observation approach was deemed insufficient, despite the absence of remarkable examination findings. An abdominal ultrasound was conducted one day after admission, which indicated minimal fluid and mild thickening of the bowel in the right iliac fossa, aligning with the patient’s site of pain but not corresponding to her external injuries. The appendix was not visualized during the ultrasound. As the patient remained stable and showed overall positive progress, observation was continued to monitor her symptoms and signs. However, on the third day of hospital admission, her symptoms persisted without improvement. Consequently, a decision was made to perform a contrast-enhanced computed tomography of the abdomen, which revealed a thickened wall of the terminal ileum with a suspicious wall defect consistent with ileal perforation. (Fig. 2A, Fig. 2B, Fig. 2C).

**Table 1:** Hematological and biochemical findings of the patient.

Examination	Result	Reference range
CBC- On day of presentation		
Total leukocyte counts	13.5 thou/ul	4.5–11.0×10^9^/l
Neutrophil	77	40–70%
Lymphocyte	18	20–45%
Monocyte	3	2–10%
Eosinophil	2	1–6%
Hemoglobin	13.4 g/dl	12–14 g/dl
RBC count	5.02 mill/cumm	4.5–5.5 mill/cumm
PCV	41	35–54
M.C.V	82 fl	80–100 fl
M.C.H	27 pg	26–34 pg
M.C.H.C	33%	32–36%
RDW	12.3%	11–16%
Platelets count	291 000 thou/ul	150 000–450 000
CBC- Day 2		
Total Leukocyte Counts	15.7 thou/ul	4.5–11.0×10^9^/l
Neutrophil	93	40–70%
Lymphocyte	5	20–45%
Monocyte	1	2–10%
Eosinophil	1	1–6%
Hemoglobin	12.8 g/dl	12–14 g/dl
RBC count	4.5 mill/cumm	4.5–5.5 mill/cumm
PCV	37	35–54
M.C.V	82 fl	80–100 fl
M.C.H	28 pg	26–34 pg
M.C.H.C	34%	32–36%
RDW	12.7%	11–16%
Platelets count	367 000 thou/ul	150 000–450 000
Serum- Day 2		
Serum Amylase	142 U/l	25–125 U/l
Serum Lipase	55 U/L	0–160 U/l
C-reactive protein	59.1 mg/l	<10 mg/l

Upon identifying the abnormalities in the computed tomography (CT) scan, a diagnosis of ileal perforation secondary to BAT was established. The patient was initiated on intravenous ceftriaxone and metronidazole for treatment. The diagnosis was communicated to both the patient and her parents, and the prognosis was explained. The urgency of surgical exploration and intervention was emphasized, leading to the patient being taken for surgery.

**Figure 3:**
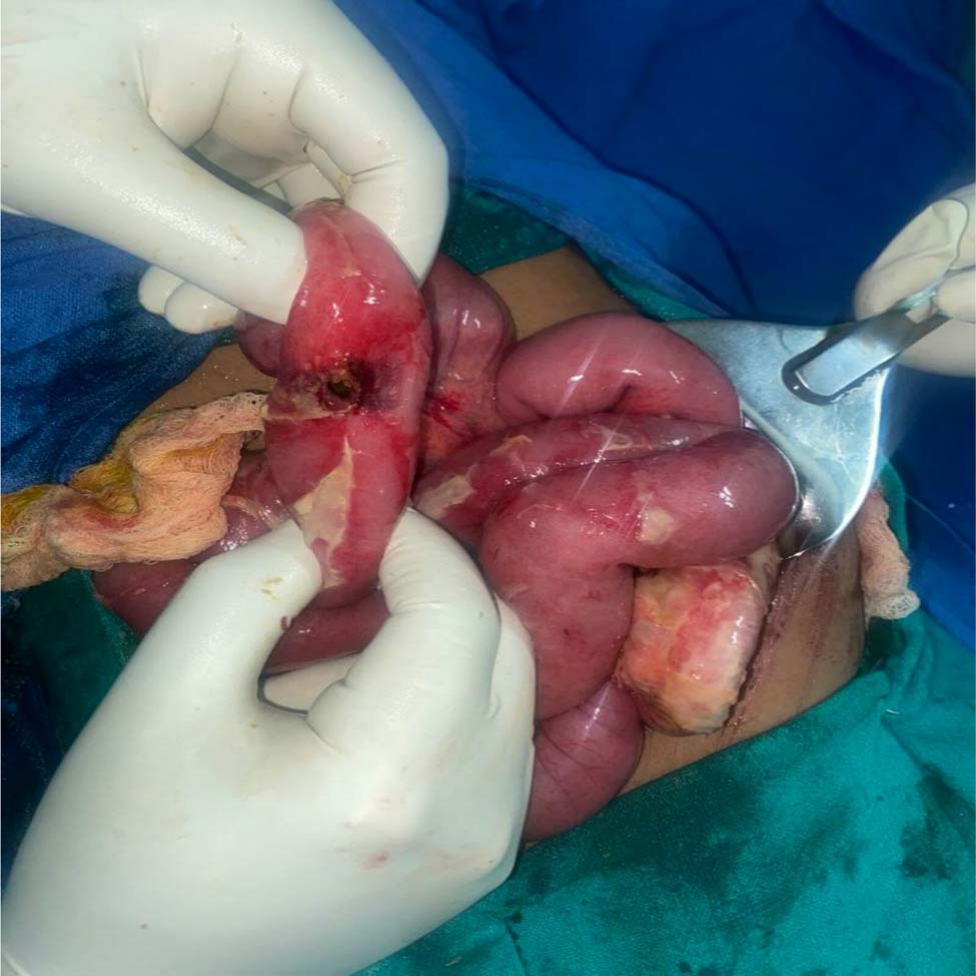
Operative view showing the perforation in the wall of the ileum.

An exploratory laparotomy was performed, which showed a 2×2 cm^2^ perforation on the ileum covered by the omentum ~25 cm from the iliocecal junction (Fig. 3). A peritoneal lavage was performed, which was followed by resection and anastomosis of the traumatic section. During the postoperative course, the patient developed some abdominal distension and was able to pass flatus but not stool. Conservative management including adjustments in her diet and regular ambulation improved her symptoms and the distension subsided. She was kept in the hospital for 4 days after which she was discharged with proper counseling on wound care, continuation of oral antibiotics, possible warning signs, diet, and follow-up visits. On follow-up after a week, the patient had a healthy wound and a good appetite. She was passing stool normally and had no complaints. The patient and her parents were initially very concerned about the potential injuries as a result of the fall. Although the symptoms were not suggestive of any grievous injury during the initial days of presentation, they were told about the delayed and possible complications of the injury, which led them to accept the diagnosis and proceed with the urgent treatment on the third day of hospital admission. They were thankful for the timely treatment and constant supervision without the need for referral to a higher center.

## Discussion

Traumatic small bowel injury is rare and presents in only 1% of patients following blunt trauma^6^. The mechanism of bowel injury in blunt trauma can differ from that of penetrating injury and hence may be responsible for atypical and/or delayed presentation. During blunt injury, a sudden increase in intraluminal pressure in a fluid or air-filled bowel loop causes punctate or slit-like perforations (blowouts) on the antimesenteric border^3^. Most of the time, these perforations are not surrounded by damaged tissue because perforation occurs due to raised intraluminal pressure and not due to crushing^3^. So, BAT causes compression necrosis of the wall of the gut, and due to high intraluminal pressure, there may be blowout perforation subsequently and hence is not necessary that BAT should cause immediate perforation^3^. The diagnostic difficulty is sometimes presented by the patient with an impaired level of consciousness and/or associated remote injuries, which may distract the clinician from the abdomen^4^. In the early hours of injury, less than 50% of the cases show free air, thus limiting the usefulness of erect chest radiography or abdomen film^2^. This was encountered in our case as well. Although the patient presented with vague abdominal pain, neither the examination findings nor the X-ray pointed toward a diagnosis of bowel perforation. As a result of which conservative management was continued for a prolonged duration.

Studies have shown that small bowel perforation if treated earlier than 12 h, then the rate of complication and mortality is low^2^. Although in our case, the patient was subsequently diagnosed with the aid of a CT and underwent timely operative intervention, a delay in diagnosis could have had a more grievous outcome. Signs and symptoms of bowel and mesenteric injuries may take up to 24–72 h to manifest^7^ .The intraoperative finding of the omentum covering the perforation and the continuous analgesic received by the patient might be the contributing factors in masking the frank signs and symptoms of peritonitis in our case. Frequent assessment of patients with such injuries is thus required. Some studies also suggest immediate laparotomy if clinical parameters deteriorate, or do not improve over a 12–18 h period^4^. As delayed perforations can occur after abdominal trauma, prolonged observation and repeated examination up to 72 h are mandatory for a proper diagnosis^2^. However, a study done in South Korea also reports that delay in surgical intervention following BAT may not affect the outcomes of patients with small bowel injury with regard to length of stay, ICU stays, morbidity, and mortality except for bowel resection^8^. Due to its high sensitivity, CT is considered the gold standard for the evaluation of suspected intra-abdominal injuries after blunt trauma in adults but doses of ionizing radiation brought by CT can damage DNA, increasing the risk of cancer throughout life and should be kept in consideration while ordering CT in children^9^. A study in Brazil concluded that although CT is necessary in some instances, there is a possible high number of exams that did not make any difference in the management of the pediatric population^9^. The FAST examination has also been associated with improvements in outcomes among adult patients including reductions in abdominal computed tomographic (CT) rates, decreased time to the operating room, and decreased hospital length of stay^10^. However, the FAST scan was negative at presentation in our case. So, the symptoms, signs along with the risk and the benefits of investigation modalities should always be taken into account. Since this is a report of a single case, further studies are required to recommend the guidelines for the management of case like ours.

Providing comprehensive counseling to both patients and their guardians regarding all potential outcomes is a crucial aspect of managing cases like this. In our specific case, the parents were highly anxious when they first brought the patient in. However, as the assessment progressed and the reports indicated findings within normal ranges, they experienced a sense of relief, despite the patient not fully recovering from her symptoms. Nevertheless, the treating doctor and the medical team maintained constant supervision over the patient and ensured that the parents were aware that she still faced potential risks. If the parents had been reassured solely based on the initial investigation reports, it would have been challenging to convince them of the need for surgery and explain the diagnosis 3 days after the initial incident. Therefore, it becomes crucial to explain to both the patients and their guardians all the potential outcomes of the event they experienced, even if it may initially seem unlikely.

## Conclusion

Small bowel perforation resulting from BAT is a seldom-seen occurrence, particularly among pediatric patients. The diagnosis of this condition poses a challenge due to its unusual presentation and the absence of specific clinical markers. Diagnostic imaging methods such as ultrasound and computed tomography play a pivotal role in detecting small bowel perforations. Timely identification and prompt surgical intervention are vital for reducing complications and enhancing patient outcomes. Thorough patient counseling is essential to ensure proper management and prevent treatment delays.

## Ethical approval

Ethical approval is not required for case reports in my institution (Patan Academy of Health Sciences, Bagmati Lalitpur) so ethical approval was exempted.

## Consent

Written informed consent was obtained from the patient for the publication of this case report and accompanying images. A copy of written consent is available for review by the Editor-in-Chief of this journal on request.

## Sources of funding

Not applicable.

## Author contribution

S.K.: conceptualization, mentor and reviewer for this case report and for data interpretation; A.G.: contributed in performing literature review, writing the paper and editing; N.J.: contributed in writing the paper. All authors have read and approved the manuscript

## Conflicts of interest disclosure

All the authors declare that they have no competing interest.

## Research registration unique identifying number (UIN)

Not applicable.

## Guarantor

Shailendra Katwal.

## Data availability statement

Not applicable.

## Provenance and peer review

Non commissioned, externally peer reviewed.
